# Degradation of Benzo[a]pyrene by *Rhodococcus* sp. PDS1 Under Combined Pollution of Arsenic and Polycyclic Aromatic Hydrocarbons

**DOI:** 10.3390/microorganisms14040811

**Published:** 2026-04-02

**Authors:** Mei-Lin Zheng, Hong-Peng Shi, Ying-Hao Zhao, Ying Liu, Luyan Ma, Zhi-Pei Liu

**Affiliations:** 1 State Key Laboratory of Microbial Resources, Institute of Microbiology, Chinese Academy of Sciences, Beijing 100101, China; zhengml@im.ac.cn (M.-L.Z.); shihongpeng21@mails.ucas.ac.cn (H.-P.S.); zhaoyh@im.ac.cn (Y.-H.Z.); liuying@im.ac.cn (Y.L.); 2 University of Chinese Academy of Sciences, Beijing 101408, China

**Keywords:** polycyclic aromatic hydrocarbons, arsenic, combined pollution, PAH bioremediation

## Abstract

Polycyclic aromatic hydrocarbons (PAHs)-contaminated soils are often concomitantly polluted with heavy metals, which form combined contamination through cation–π interactions and other mechanisms. However, the mechanism by which bacteria degrade PAHs under combined pollution conditions remains insufficiently studied. In this study, a benzo[a]pyrene (BaP)-degrading bacterial strain, *Rhodococcus* sp. PDS1, was isolated from the co-contaminated soil of an abandoned coking plant in a steel factory. This strain can not only detoxify arsenic via reductive transformation, but also mediate extracellular arsenic oxidation and efficiently degrade BaP, a high-molecular-weight (HMW) polycyclic aromatic hydrocarbon with low bioavailability and high toxicity. Response surface methodology (RSM) experiments were conducted to optimize the degrading conditions of strain PDS1, considering four factors: pH, temperature, BaP concentration, and trivalent arsenic As(III) concentration. The results showed that the BaP removal by PDS1 would reach 93.59% under the RSM-obtained optimal conditions: pH 7.7, BaP concentration 8.96 mg/L, As(III) concentration 0.82 mM, and culture temperature 36.0 °C. The transcriptome of the strain under the combined stress of arsenic and BaP was further analyzed. The results indicated that the introduction of arsenic induced the upregulated expression of different genes in the arsenic detoxification *ars* operon and the *pcaH/G* gene (encoding protocatechuate 3,4-dioxygenase, a key enzyme in BaP degradation) to varying degrees. These findings clarify the mechanism of the degradation of HMW-PAHs such as BaP by strain PDS1 under PAHs–arsenic combined pollution, lay a solid theoretical foundation for subsequent practical applications, and demonstrate the broad prospects of strain PDS1 in the remediation of actual complex contaminated soils.

## 1. Introduction

Polycyclic aromatic hydrocarbons (PAHs) are hydrocarbons in which two or more benzene rings are connected in a polycyclic structure [1]. As a common category of persistent organic pollutants in the environment, they have long been a focus of attention for the scientific community and government decision-making bodies [2,3,4]. This is because of their carcinogenic, teratogenic, and mutagenic properties, along with their capacity for long-distance migration. Although some natural processes also produce PAH emissions [5], such as volcanic eruptions, PAHs in the environment predominantly derive from anthropogenic activities, encompassing combustion-related processes such as fossil fuel combustion and waste incineration, alongside a spectrum of industrial manufacturing operations including electrolytic aluminum smelting, coking, and petroleum refining [6]. In the list of 126 priority control pollutants issued by the U.S. National Environmental Protection Agency (EPA), there are 16 PAHs, accounting for about 1/8 [7]. Among these, benzo[a]pyrene (BaP), which consists of five benzene rings, is the first discovered environmental chemical carcinogen [8], and one of the high-molecular-weight PAHs (HMW-PAHs) with the widest distribution, strongest toxicity, highest risk of carcinogenicity, and the greatest difficulty in degradation [9]. BaP can be adhered to aerosols and migrate in the flow of atmospheric environment, and it can also enter the soil and water bodies through dry and wet deposition [1], resulting in the contamination of different environmental matrices [10]. Insolubility and lipophilicity make BaP preferentially adsorbed to soil and organic matter-rich particles, causing impairment of soil ecosystem functions. BaP can be absorbed by the human body through inhalation, dermal contact, and bioaccumulation, and by generating carcinogenically active metabolites and disrupting DNA replication [11], it increases the incidence of lung cancer [12], skin cancer [13], and gastric cancer, posing a severe threat to human health [14].

Arsenic (As) is a metalloid that naturally occurs in the Earth’s crust and is widely distributed in environmental matrices such as water, soil, and sediment [15,16]. However, due to its chemical properties, it is classified as a heavy metal (HMs). Natural processes such as pedogenesis, volcanic eruptions, and weathering and leaching of sediments are the primary sources of arsenic. However, the main causes of arsenic pollution are human activities such as mining, metal smelting, industrial wastewater, and excessive use of arsenic-containing pesticides [17]. The results of the national soil pollution survey [18] indicate that the overall over-standard rate of soil in China is 16.1%, and the number of points exceeding the standard for inorganic pollutants accounts for 82.8% of the total number of over-standard points. A total of approximately 2.5 million square kilometers of cultivated land, forest land, and grassland have been contaminated by inorganic pollutants, mainly arsenic, cadmium, and nickel. In some typical plots surveyed, such as areas along trunk roads, sewage irrigation districts, mining areas, metallurgical industrial parks, and abandoned construction sites, varying degrees of arsenic pollution have also been detected. Overall, the distribution of arsenic content shows a gradual increasing trend from northwest to southeast and from northeast to southwest in China [19,20]. Arsenic exists in the environment in four main chemical forms: arsenate As(V), arsenite As(III), elemental arsenic (As), and arsenide As(III−) [21]. Inorganic arsenic is classified as a Group 1 carcinogen capable of inducing a variety of cancers. Of its different chemical species, trivalent arsenic exhibits significantly higher toxicity, being approximately 10-fold more toxic than pentavalent arsenic [22]. Accordingly, inorganic arsenic is listed as the top priority contaminant on the priority list of hazardous substances by the United States Environmental Protection Agency (EPA), the Agency for Toxic Substances and Disease Registry (ATSDR), and the International Agency for Research on Cancer (IARC) [23].

PAHs and HMs frequently coexist in the soil at sites such as chemical industrial parks [24], waste incineration and landfills [25], mines [26], and zones adjacent to arterial highways [27]. These contaminants undergo complex and diverse interactions, including cation–π interactions [28], competition and synergy for adsorption sites [29,30], and redox reactions [31], thereby forming combined pollution. The toxic effects of such co-contamination are significantly greater than the sum of their individual impacts, posing more severe threats to ecosystems and human health while increasing the complexity of related research and remediation. Compared with chemical and physical remediation, microbial remediation offers higher economic efficiency and better sustainability. Furthermore, its environmentally friendly properties endow it with broader prospects in environmental remediation practices, gradually making it a focus of research attention [32]. To date, there have been relatively few reports on bifunctional strains capable of both PAH degradation and HM transformation, and their underlying mechanisms of action remain poorly understood. In our previous work, therefore, a bacterial strain isolated from the contaminated soil, *Rhodoccocus* sp. 2021, was able to efficiently degrade fluorene under the combined pollution of fluorene and arsenic, and arsenic could greatly improve its degrading ability [33]. Herein, another bacterial strain, *Rhodoccocus* sp. PDS1 capable of degrading BaP under the combined pollution of Bap and As(III), was characterized for its Bap-degrading ability, including the optimization of the degradation conditions via response surface methodology and decoded the regulatory mechanisms through transcriptome sequencing. A hypothesis was proposed that microbes mediate the interaction among organic pollutants and inorganic pollutants. This study provides critical scientific insights for the development of remediation technologies against PAHs-As composite pollution, underscoring its high academic and application value.

## 2. Materials and Methods

### 2.1. Main Chemicals and Culture Medium

Benzo[a]pyrene (BaP, analytical grade, OKA, Beijing, China) was used as the substrate in degradation tests or as otherwise indicated. Sodium arsenite (analytical grade, Sigma-Aldrich, St. Louis, MO, USA) was used as the trivalent arsenic compound for subsequent tests. Disodium hydrogen arsenate (analytical grade, Sigma-Aldrich, St. Louis, MO, USA) was used as the pentavalent arsenic compound for subsequent tests. All other reagents were available in the laboratory unless otherwise specified. BaP was dissolved in acetone to prepare a 5 g/L stock solution, which was then filtered through a 0.22 μm sterile polytetrafluoroethylene (PTFE) filter and stored at 4 °C for subsequent use. Sodium arsenite and disodium hydrogen arsenate were dissolved in distilled water to prepare 2 M and 5 M stock solutions, respectively. After being filtered through a 0.22 μm sterile polyethersulfone (PES) filter, the solutions were stored at 4 °C for subsequent use.

The mineral salt medium (MSM) contained (g/L): KH_2_PO_4_: 1.5, Na_2_HPO_4_·12H_2_O: 0.5, NaCl: 1.0, (NH_4_)_2_SO_4_: 1.0, MgSO_4_·7H_2_O: 0.06, yeast extract: 0.05, and trace element solution: 2 mL. When needed, 2.68 g of mannitol is added to each liter of culture medium as an additional carbon source. And this medium is denoted as DM-MSM. Trace element solution contained (g/L): FeSO_4_·7H_2_O: 0.5, ZnSO_4_·7H_2_O: 0.01, MnCl_2_·4H_2_O: 0.003, H_3_BO_3_: 0.03, CoCl_2_·6H_2_O: 0.02, CuCl_2_·2H_2_O: 0.0001, NiCl_2_·6H_2_O: 0.002, and Na_2_MoO_4_·2H_2_O: 0.003. Luria–Bertani medium (LB) (g/L): peptone: 10, yeast extract: 5, and NaCl: 10. To prepare agar plates, 1.5% agar was added to the medium.

### 2.2. Screening, Isolation and Identification of Degrading Strain

The soil samples for strain screening were collected from a contaminated site of a coking plant in Hangzhou, China. The site has long been subjected to combined pollution by polycyclic aromatic hydrocarbons, petroleum hydrocarbons and heavy metals, among which the pollution level of BaP is 42.16 ± 8.21 mg/kg, and that of total As is 99.51 ± 8.22 mg/kg [24]. A total of three soil layers at different depths were collected, specifically 0–10 cm, 10–30 cm, and 30–60 cm. For each depth, the collected samples were first subjected to preliminary homogenization after removing gravel and plant residues. Subsequently, 5 subsamples (10 g each) were weighed from the soil samples of each depth, and all these subsamples were homogenized again. Then, 10 g of the homogenized sample was weighed and added to a 250 mL sterile conical flask containing 90 mL of sterile normal saline. The flask was placed on a shaker, incubated at 30 °C~180 rpm for 2 h, and then taken out for standing to prepare a soil suspension. BaP stock solution was added to a dried 250 mL sterile conical flask. Once acetone had completely volatilized, 90 mL of MSM was added. The conical flask was then placed in an ultrasonic cleaner for sonication until BaP solids detached from the inner wall of the conical flask and were fully suspended in the liquid. 10 mL of the soil suspension was inoculated into the conical flask, followed by the addition of sodium arsenite stock solution to achieve a final arsenic concentration of 0.1 mM. The final concentration of BaP was 2 mg/L. After 2 weeks of shaking culture at 30 °C and 180 rpm, 20% (*v*/*v*) of the bacterial suspension was transferred to a fresh MSM with a higher concentration of sodium arsenite (0.5 mM) and a higher concentration of benzo[a]pyrene (5 mg/L). The above operation was repeated until the maximum concentration of sodium arsenite reached 2 mM and the maximum concentration of BaP reached 20 mg/L. Under this condition, subculturing was performed repeatedly until a stable microbial community was obtained.

After serial dilution of the culture, the diluted suspension was spread onto MSM agar plates containing 10 mg/L BaP and 1 mM sodium arsenite, and then incubated in a 30 °C incubator. Once colonies grew, single colonies were picked and inoculated into MSM containing 10 mg/L BaP and 1 mM sodium arsenite to observe whether the strains could grow. Strains that were able to grow were streaked and purified using LB agar plates. The 16S rRNA gene of the purified bacterial strain was amplified via PCR using the universal primer pair 27F (5′-AGAGTTTGATCCTGGCTCAG-3′) and 1492R (5′-GGTTACCTTGTTACGACTT-3′) [34]. The amplified PCR products were sequenced by Majorbio Bio-Pharm Technology Co., Ltd. (Shanghai, China). For taxonomic identification, the resulting sequences were subjected to a homology search against the National Center for Biotechnology Information (NCBI) nucleotide collection database using the Basic Local Alignment Search Tool (BLAST, https://blast.ncbi.nlm.nih.gov/Blast.cgi?PROGRAM=blastn&PAGE_TYPE=BlastSearch&LINK_LOC=blasthome, accessed on 8 March 2026).

The isolated strains were individually inoculated into mineral salt medium (MSM) containing 20 mg/L benzo[a]pyrene and 2 mM sodium arsenite, with an initial pH of 7.0. Three experimental replicates were set up for each strain, and the same system without inoculated strains served as the control group. The cultures were incubated at 30 °C with shaking at 120 rpm for 14 days, after which the residual polycyclic aromatic hydrocarbon (PAH) content in the culture system was detected to determine the benzo[a]pyrene removal efficiency of each strain. The strain with the highest removal rate was selected for subsequent studies.

### 2.3. Optimization of Strain Culture Conditions

This study adopted the principle of Box–Behnken design (BBD) [35] to conduct a response surface optimization experiment with four factors and three levels. The following four factors were selected as independent variables, namely: A (pH value), B (BaP concentration), C (As(III) concentration), and D (temperature); the BaP removal rate (%) was used as the response value (Y). The coding of experimental factors and levels is shown in Table 1. According to the Box–Behnken design, a total of 29 experimental points were included to evaluate the main effects and interaction effects of each factor, and a response surface regression model was established. Design-Expert 13.0.1.0 software was used to perform quadratic multiple regression analysis on the experimental data, and a regression equation was obtained by fitting. One-way analysis of variance (ANOVA) was used to test the significance and lack of fit of the model, while the coefficient of determination (R^2^) and adjusted coefficient of determination (R_adj_^2^) were used to evaluate the fitting effect of the model. Origin 2022 software was employed to draw three-dimensional response surface plots and contour plots.

The strain was inoculated into DM-MSM and cultured in a constant temperature shaker at 37 °C with shaking at 180 rpm. When the strain grew to the mid-logarithmic growth phase, the bacterial cells were collected by centrifugation at 12,000 rpm for 2 min. After washing the bacterial cells three times, they were resuspended in MSM, and OD_600_ of the bacterial suspension was adjusted to 1 to prepare the seed solution. Subsequently, inoculation was carried out at a volume ratio of 1%. All experiments were set with 3 parallel replicate samples. After culturing at 37 °C with shaking at 180 rpm for 3 weeks, the residual amount of BaP in the system was determined.

### 2.4. Arsenic Valence Transformation

The strain was inoculated into MSM supplemented with 1 mM sodium arsenite and 10 mg/L benzo[a]pyrene, with an initial pH of 8. The inoculated group was set as the experimental group (S), while the non-inoculated group served as the control group (CK), both with three biological replicates. All cultures were incubated at 37 °C with shaking at 180 rpm for 8 weeks, and samples were collected weekly during cultivation. After filtration through 0.22 μm membrane filters, the concentration of pentavalent arsenic was determined using an ion chromatography system (ICS-2100, Thermo Fisher Scientific, Waltham, MA, USA).

### 2.5. Analytical Methods

The growth of a bacterial strain was monitored by measuring the density of the cultures at λ = 600 nm (OD_600_) using a UV–visible spectrophotometer (UV-VIS 2008, UNICO Instrument Co., Ltd., Shanghai, China). The pH was determined using a handheld multi-parameter meter (WTW Multi 3420, Xylem Analytics Germany Sales GmbH & Co. KG, Weilheim, Bavaria, Germany).

BaP was recovered from the medium via liquid–liquid extraction with an extraction solvent composed of ethyl acetate and n-hexane at a volume ratio of 1:1. Ultrasonic extraction was performed 4 times, and the organic phases were combined into a distillation flask. The mixture was concentrated to less than 1 mL using a rotary evaporator at 35 °C under 0.08 MPa, after which the solvent was replaced with acetonitrile and the volume was adjusted to the required level in a volumetric flask. Prior to injection, the sample was be filtered using a 0.22 μm organic nylon filter. BaP was quantified by high-performance liquid chromatography (HPLC, Agilent 1260 Infinity II, Agilent Technologies, Santa Clara, CA, USA) with a chromatographic column (Extend-C18, 4.6 × 250 mm, 5 μm, Agilent Technologies, Santa Clara, CA, USA). The mobile phase consisted of 85% acetonitrile and 15% ultrapure water (containing 0.1% formic acid). The detector used was an Agilent 1260 Infinity II variable wavelength detector. The detection wavelength was 295 nm; the injection volume was 10 μL; the mobile phase flow rate was 1 mL/min; and the column temperature was 25 °C.

Arsenic speciation analysis was conducted using anion liquid chromatography (ICS-2100, Thermo Fisher Scientific, Waltham, MA, USA) using an anion chromatographic column (PP, 4 × 250 mm, 5 μm, 046129, Thermo Fisher Scientific, Waltham, MA, USA). The eluent was potassium hydroxide; the flow rate was 1 mL/min, and the column temperature was 30 °C. The gradient elution program was set as follows: 0–5 min, potassium hydroxide concentration at 20 mmol/L; 5–15 min, linear increase to 50 mmol/L; 15–15.1 min, potassium hydroxide concentration ramped up to 60 mmol/L and held until 20 min; 20–20.1 min, potassium hydroxide concentration dropped to 20 mM and held until 25 min. The culture broth was centrifuged at 12,000 rpm, and the supernatant was harvested. After filtration through a 0.22 μm aqueous membrane filter, the sample was injected with an injection volume of 25 μL.

### 2.6. Transcriptome Analysis

To harvest a sufficient quantity of bacteria for the completion of total RNA extraction, bacterial strain was cultured in DM-MSM, with three experimental groups set up as follows: group control check (CK) (DM-MSM + strain), group BaP (DM-MSM + strain + 5 mg/L BaP), and combined group BaP-As(III) (DM-MSM + strain + 5 mg/L BaP + 1 mM sodium arsenite). Strain colonies were scraped from LB plate (1 mM arsenite), rinsed three times with sterile water, and resuspended for about half an hour. The suspension was briefly standing to precipitate large cell clumps, and the upper homogeneous fraction was aspirated, adjusted to an optical density (OD) of 1.0, and inoculated into conical flasks at a ratio of 1% (*v*/*v*). Cultures were incubated at 37 °C with shaking at 200 rpm until reaching the mid-logarithmic growth phase, after which cells were harvested. Total RNA was extracted using TRIzol Reagent (Invitrogen, Carlsbad, CA, USA), and genomic DNA contamination was eliminated by treatment with DNase I (Takara Bio Inc., Kusatsu, Shiga, Japan). RNA concentration and purity were determined using a NanoDrop 2000 spectrophotometer (Thermo Fisher Scientific, Waltham, MA, USA), RNA integrity was verified by agarose gel electrophoresis, and the RNA Integrity Number (RIN) was measured with an Agilent 2100 Bioanalyzer (Agilent Technologies, Santa Clara, CA, USA) to ensure all samples met the criteria for library construction.

Transcriptome libraries were constructed using the TruSeq™ Stranded Total RNA Library Prep Kit (Illumina Inc., San Diego, CA, USA). Ribosomal RNA (rRNA) was depleted using the Ribo-Zero Magnetic Kit (Epicentre, an Illumina company, Madison, WI, USA), and the remaining mRNA was fragmented to approximately 200 bp. First-strand cDNA was synthesized with random hexamer primers, and dUTP was substituted for dTTP during second-strand synthesis, resulting in the second cDNA strand containing adenine (A), uracil (U), cytosine (C), and guanine (G). The second cDNA strand was then digested with uracil-N-glycosylase (UNG), ensuring that only the first cDNA strand was retained in the library. After quantification with a TBS-380 fluorometer (Turner BioSystems Inc., Sunnyvale, CA, USA) and PCR amplification, the libraries were sequenced on the Illumina HiSeq × Ten platform (Shanghai Majorbio Bio-Pharm Technology Co., Ltd., Shanghai, China).

Sequencing data were analyzed using the Majorbio Cloud Platform. Gene expression levels were quantified as Transcripts Per Million mapped reads (TPM). Differentially expressed genes (DEGs) were identified using the edgeR 4.0.2, NOISeq 2.46.0, and DESeq 1.56.1 software packages. Gene Ontology (GO) functional enrichment analysis and Kyoto Encyclopedia of Genes and Genomes (KEGG) pathway enrichment analysis were performed using Goatools 1.4.1 and KOBAS 2.0, respectively, to screen for statistically significant enriched pathways.

## 3. Results

### 3.1. Isolation and Identification of Benzo[a]pyrene-Degrading Strain

**Figure A1:**
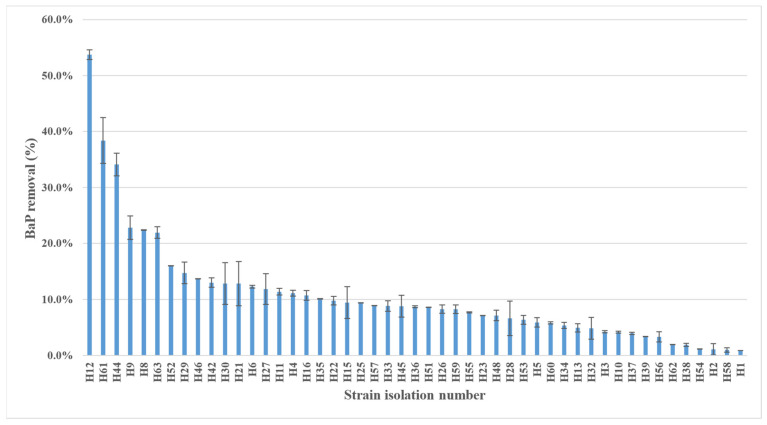
Preliminary determination of BaP removal rate by the strains. Error bars represent the standard deviation (SD) of three independent experiments (*n* = 3).

Ultimately, after several successive subcultures in mineral salt medium using BaP as the substrate, a stable bacterial community was successfully obtained. This community was able to grow under the conditions where benzo[a]pyrene served as the sole carbon and energy source, and sodium arsenite as the stress factor. Strains belonging to more than ten genera were isolated from this community (Table A1), among which the strain numbered H12 showed the highest benzo[a]pyrene removal efficiency under the same conditions (Figure A1).

For preliminary identification of this strain, a fragment with 1393 bp of its 16S rRNA gene was obtained by PCR as mentioned above. BLAST analysis against the GenBank database revealed that it shared the highest sequence similarity (99.6%) with *Rhodococcus aetherivorans* (accession number JN180179.1). Therefore, the strain was identified as a member of the genus *Rhodococcus*, and named *Rhodococcus* sp. PDS1. This strain has been deposited in the China General Microbiological Culture Collection Center (CGMCC), with the deposit registration number CGMCC 1.65083.

### 3.2. Effects of Physical and Chemical Factors on the Removal of BaP by Strain PDS1

#### 3.2.1. Establishment of the Box–Behnken Regression Model

A total of 29 experiments were conducted following the Box–Behnken design, incorporating four factors (BaP concentration, As(III) concentration, pH, and temperature) with the BaP removal rate as the response variable. Significance analysis and analysis of variance (ANOVA) were performed on the experimental data obtained via response surface methodology (RSM), yielding the following regression Equation (1):

(1) Y = −150.91 + 43.84A + 2.46B + 9.81C + 3.19D − 0.72AB −0.68AC + 1.16AD + 0.22BC+ 0.12BD + 0.09CD − 4.75A^2^ − 0.10B^2^ − 4.72C^2^ − 0.20D^2^.

Y is the response value, i.e., BaP removal efficiency. A is pH value, B is BaP concentration, C is As(III) concentration, and D is temperature. AB, AC, AD, BC, BD, and CD represent the interaction terms between pH value and BaP concentration, pH value and As(III) concentration, pH value and temperature, BaP concentration and As(III) concentration, BaP concentration and temperature, and As(III) concentration and temperature, respectively. A^2^, B^2^, C^2^, and D^2^ represent the quadratic terms of pH value, BaP concentration, As(III) concentration, and temperature, respectively.

As shown in Table 2, the regression equation generated an F-value of 45.33, a coefficient of determination (R^2^) of 0.9784, and a *p* < 0.0001, indicating that 97.84% of the variation in the response variable can be explained by this model. The coefficient of variation (C.V.) was 10.54%, confirming the feasibility of using RSM to analyze the effects of physicochemical factors on BaP removal by strain PDS1, as well as to establish and predict the model.

In the quadratic regression model, the quadratic coefficients for all four factors—pH (A), BaP concentration (B), As(III) concentration (C), and temperature (D)—were negative, indicating that the model contains stable vertices for these factors, corresponding to a maximum BaP removal rate under the experimental conditions. Additionally, the lack-of-fit term in the model yielded an F-value of 3.87 and a *p*-value of 0.1022, suggesting non-significant lack-of-fit error, i.e., the experimental error was relatively small. The processed data from the response surface experiments demonstrated that the regression equation derived via RSM had a high goodness of fit with a significant regression effect, confirming the feasibility and accuracy of the experimental results. Thus, the multiple regression relationship between the independent variables and the response variable is applicable to this model.

Based on the F-values and *p*-values presented in Table 2, among the linear terms, BaP concentration (B) and As concentration (C) exerted significant effects (*p* < 0.01), whereas pH (A) and temperature (D) showed no significant effects. The order of influence of the four factors on the BaP removal was determined as follows: As(III) concentration (C) > BaP concentration (B) > pH (A) > temperature (D). For the interaction terms among the four factors, the AC, BC, and CD interactions had no significant impact on BaP removal. In contrast, the AB and AD interactions exerted extremely significant effects (*p* < 0.01), and the BD interaction had a significant effect (*p* < 0.05), with the order of influence being AB > AD > BD.

#### 3.2.2. Analysis and Optimization of Conditions for BaP Removal by PDS1 Using RSM

Two of the four factors were designated as the X-axis and Y-axis, respectively, with the BaP removal efficiency serving as the Z-axis. For each calculation, the other two factors are fixed at their respective middle levels, while the remaining two are treated as variables. The middle levels of A, B, C, and D are 8, 10.25, 2.5, and 36, respectively. Three-dimensional surface plots were constructed using Origin 2022 SR1 9.9.0.225 software, which enables intuitive visualization of how interactive effects influence BaP removal performance. The color gradient of the plots, ranging from blue to red, corresponds to the magnitude of response values, with blue representing lower values and red indicating higher ones. The rate of color transition reflects the changing rate of response values, i.e., the slope gradient. A steeper slope signifies a more pronounced influence within that specific region. Contour plots illustrate the extent of interaction between variables through their shapes. Contours approximating a circular shape indicate less significant interaction between the two variables, whereas elliptical contours suggest more prominent interactive effects. Regions with denser contour lines correspond to greater variations in response values, while sparser contours denote more gradual changes.

**Figure 1:**
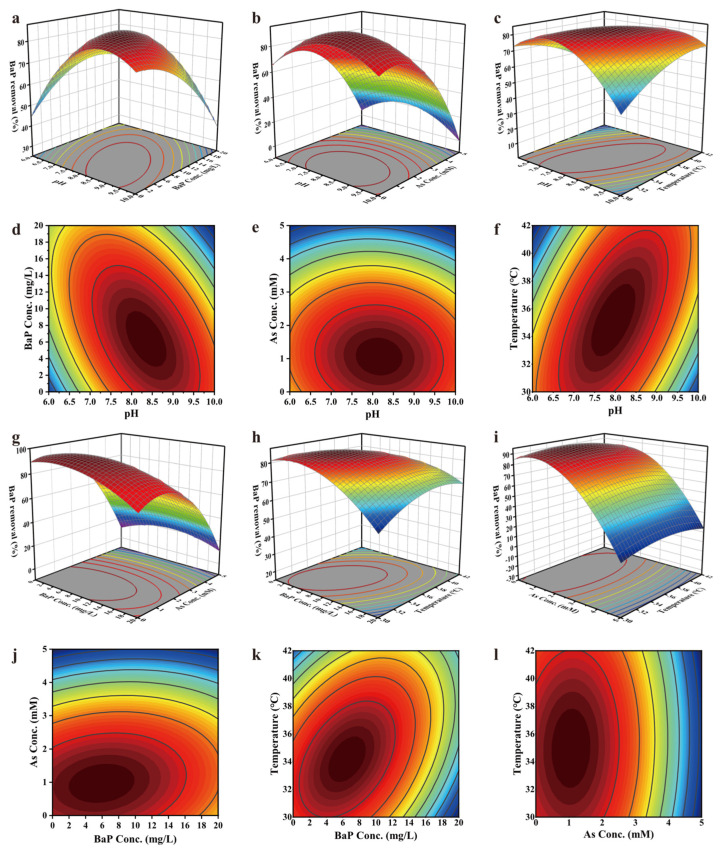
Response surface plots of the effect of physical and chemical factors on the removal of BaP by strain PDS1. Note: The four factors investigated in the experiment are pH value (A), BaP concentration (B), trivalent arsenic concentration (C), and Temperature (D). The response value is the removal rate of BaP, and the culture time is 1 month. (**a**) Three-dimensional response surface plot of the interaction between A and B; (**b**) Contour plot of the interaction between A and B; (**c**) Three-dimensional response surface plot of the interaction between A and C; (**d**) Contour plot of the interaction between A and C; (**e**) Three-dimensional response surface plot of the interaction between A and D; (**f**) Contour plot of the interaction between A and D; (**g**) Three-dimensional response surface plot of the interaction between B and C; (**h**) Contour plot of the interaction between B and C; (**i**) Three-dimensional response surface plot of the interaction between B and D; (**j**) Contour plot of the interaction between B and D; (**k**) Three-dimensional response surface plot of the interaction between C and D; (**l**) Contour plot of the interaction between C and D. The color scheme in the three-dimensional surface plots and contour maps denotes the level of BaP removal efficiency, where the color intensifies progressively from blue to red, corresponding to a continuous rise in BaP removal efficiency.

As shown in Figure 1a, the BaP removal efficiency first increased from 44.20–82.37% to 66.20–85.44% with increasing BaP concentration from 0 to 20 mg/L, and then decreased to 36.95–71.31%, showing a trend of increase followed by decrease. A similar pattern was observed with increasing pH from 6 to 10, where the BaP removal efficiency rose from 44.20–66.20% to 71.31–85.44% and then declined to 36.96–75.96%. The elliptical and dense contour lines indicated a significant interaction between BaP concentration (B) and pH (A). The higher contour density along the BaP concentration axis suggested that the effect of BaP concentration was more significant than that of pH (Figure 1b).

As shown in Figure 1c, the BaP removal efficiency first increased from 4.35–72.05% to 21.02–94.02% with increasing pH, and then decreased to 0–77.99%. Similarly, with increasing As(III) concentration, the BaP removal efficiency increased from 65.10–88.76% to 72.05–94.02%, and then decreased to 0–21.02%. Both variables exhibited a trend of initial increase followed by decrease. The contour lines were nearly circular, indicating an insignificant interaction between pH (A) and As(III) concentration (C). The contour density was distinctly higher along the As(III) concentration axis, suggesting that the effect of As(III) concentration (C) on BaP removal efficiency was significantly stronger than that of pH (A) (Figure 1d).

As shown in Figure 1e, when the temperature gradually increased within the range of 30–42 °C, the BaP removal efficiency rose from 46.90–80.99% to 71.72–84.38%, showing an overall upward trend with increasing temperature and finally leveling off. As the pH value gradually increased from 6 to 10, the BaP removal efficiency first increased from 41.60–72.71% to 78.47–84.38% and then decreased to 46.90–71.62%, exhibiting a trend of initial increase followed by decline. The contour lines were elliptical and relatively dense for both factors, indicating a significant interaction between pH (A) and temperature (D). The contour density was higher along the pH axis, suggesting that the effect of pH (A) was more pronounced than that of temperature (D) (Figure 1f).

As shown in Figure 1g, with the increase in BaP concentration, the BaP removal efficiency gradually increased from 10.99–92.27% to 20.97–95.81%, and then decreased to 11.19–75.83%, showing a trend of initial increase followed by decrease. With the increase in As concentration, the BaP removal efficiency increased from the initial 67.82–91.49% to 75.83–95.81%, and then decreased to 10.00–20.97%, exhibiting a downward trend. As can be seen from the contour plot, the contour density along the As(III) concentration gradient was significantly higher than that along the BaP concentration gradient, indicating that under the interaction of the two factors (B and C), the effect of As(III) concentration (C) on BaP removal efficiency was significantly greater than that of BaP concentration (B) (Figure 1h).

As shown in Figure 1i, at the same temperature, when the BaP concentration ranged from 0 to 20 mg/L, the BaP removal efficiency first increased from 62.89–81.93% to 75.61–85.56% and then decreased to 56.51–70.08%, showing an overall decreasing trend with increasing concentration. Conversely, with increasing temperature, the BaP removal efficiency gradually increased from 56.51–82.73% to 70.08–85.56% and then leveled off. As depicted in Figure 1j, the contour map exhibited an elliptical shape with relatively high contour line density, indicating a significant interaction between these two factors. Along the BaP concentration gradient, the contour line density was higher, demonstrating that the influence of BaP concentration (B) on the BaP removal rate was greater than that of temperature (D).

As shown in Figure 1k, when the As(III) concentration was in the range of 0–5 mM, the BaP removal efficiency gradually increased from an initial 78.33–88.84% to 84.39–94.13% with the elevation of As(III) at low concentrations. With further increase in As(III) concentration, the BaP removal efficiency started to decrease significantly, dropping to 13.66–20.96% at 5 mM As(III). When the temperature increased gradually from 30 to 42 °C, the BaP removal efficiency rose from 14.04–89.28% at 30 °C to 20.96–94.13%, and then decreased to 13.66–84.39%. Overall, the BaP removal efficiency was less affected by temperature and only fluctuated slightly. Along the As(III) concentration gradient, the contour line density showed greater variation, indicating that the influence of As(III) concentration (C) was significantly greater than that of temperature (D) (Figure 1l).

In summary, the effects of the four factors—pH value (A), BaP concentration (B), As(III) concentration (C), and temperature (D)—as well as their pairwise interactions on the BaP removal rate, were consistent with the predictions of the regression model. Through response surface analysis, with maximizing the BaP removal rate as the objective, the optimal culture conditions were determined as follows: pH 7.73, BaP concentration 8.96 mg/L, As(III) concentration 0.82 mM, and culture temperature 36 °C, with a predicted BaP removal rate of 93.59%.

### 3.3. Arsenic Valence Transformation by Strain PDS1

**Figure 2:**
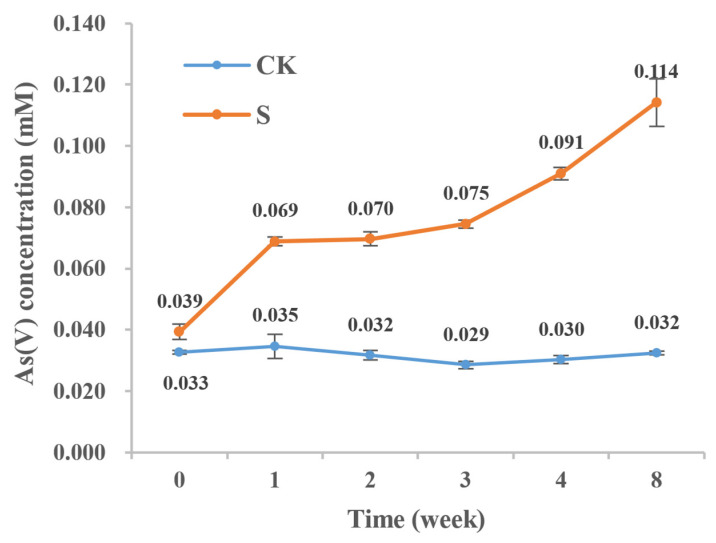
The amount of pentavalent arsenic produced in the cultivation system. In the culture system, 1 mM sodium arsenite was added. The CK group was not inoculated with the strain, while the S group was inoculated with strain PDS1.

PDS1 was inoculated and cultured in a mineral salt medium supplemented with 1 mM sodium arsenite. Samples were collected at weekly intervals, filtered through a 0.22 μm filter, and the concentration of pentavalent arsenic was determined by anion-exchange chromatography. As shown in Figure 2, the concentration of pentavalent arsenic in the control group remained relatively stable at the initial level during 0–8 weeks of incubation. In contrast, the pentavalent arsenic concentration in the experimental group increased continuously with incubation time, rising from an initial 0.039 mM to 0.114 mM. This indicates that about 10% of trivalent arsenic in the system was converted into pentavalent arsenic.

### 3.4. Transcriptomic Analysis

#### 3.4.1. Characterization of DEGs Under Different Treatments

**Figure 3:**
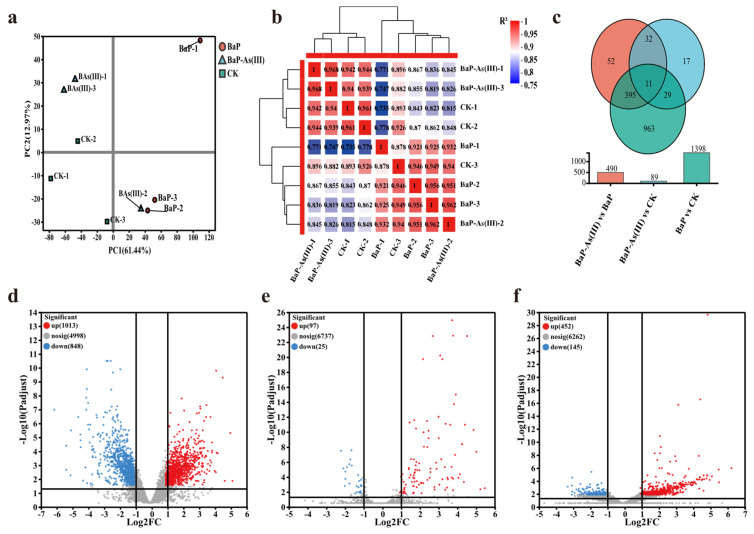
Overviews of the functional gene transcriptome changes among the three treatments. (**a**) PCA score plot of three treatments [CK, BaP, and BaP-As(III)]; (**b**) Correlation coefficient of samples based on gene expression: Numbers in the box indicated Pearson correlation coefficient between III samples; (**c**) Venn diagram of DEGs among the BaP vs. CK, BaP-As(III) vs. CK and BaP-As(III) vs. BaP comparison groups.; (**d**) Volcano plot for DEGs between BaP and CK treatments; (**e**) Volcano plot for DEGs between BaP-As(III) and CK treatments; (**f**) Volcano plot for DEGs between BaP-As(III) and BaP treatments. The upregulated and downregulated genes are highlighted with red dots and blue dots, respectively. The X-axis represents the log2-transformed expression changes, and the Y-axis represents the −log10-transformed *p*-value denoting the significance of differential expression. Probe sets with an adjusted *p* < 0.05 and a log2(|fold change|) greater than 2 between two comparison groups were defined as significant differentially expressed.

Three treatment groups [CK, BaP, BaP-As(III)] were established for transcriptome sequencing analysis. After quality control, each library obtained more than 20 million clean reads, with the average Q20 and Q30 values exceeding 97% and 93%, respectively, indicating that both the sequencing depth and data quality met the experimental requirements. The reference genome was aligned against databases including NR, Swiss-Prot, Pfam, COG, GO, and KEGG to obtain biological functional annotations of genes. Based on the gene expression profile data, principal component analysis (PCA) was performed to evaluate the correlation among samples (Figure 3a). The results showed that the variance contribution rates of PC1 and PC2 were 62.3% and 17.8%, respectively, with a cumulative contribution rate of 79.1%. This indicated good experimental reproducibility and that the PCA results could effectively reflect the overall differences among samples. Meanwhile, the three groups of samples showed a separation trend along the PC1 axis: the BaP group was significantly separated from the CK group, and the BaP-As(III) group also showed clear differentiation from the BaP group. These findings suggested that both BaP single stress and BaP-As(III) combined stress significantly regulated the gene transcription level of strain PDS1 and induced distinct expression patterns.

Pearson correlation coefficients were calculated based on the gene expression levels (TPM values) of all samples, and a sample correlation heatmap was generated (Figure 3b). The results showed that except for two samples [BaP-As(III)-2 group and CK-3] which had relatively low correlation coefficients with other samples in their respective groups, the correlation coefficients among samples in the remaining groups were high, confirming the reliability of the data. In terms of inter-group correlation, samples from the CK group, BaP single stress group, and BaP-As(III) combined stress group each formed independent clusters, and the inter-group correlation coefficients were all lower than 0.70, which were significantly lower than the intra-group correlation coefficients. This result demonstrated that both BaP single stress and BaP-As(III) combined stress significantly altered the gene transcription profile of strain PDS1, and there were obvious differences in the regulatory effects of the two stress treatments on the gene expression of the strain. In addition, the analysis results of the correlation heatmap were highly consistent with the conclusions of PCA, which further confirmed the specificity of the transcriptome expression profile of strain PDS1 under different stress treatments and provided strong support for the reliability of subsequent DEGs screening and functional enrichment analysis.

Venn diagrams and bar charts illustrated the distribution and quantitative characteristics of DEGs in the bacterial strain under CK, BaP, and BaP-As(III) treatments (Figure 3c). Only 11 DEGs were shared across all three treatment comparisons, indicating a scarcity of universally responsive genes across the different stress conditions. The BaP vs. CK comparison yielded the highest number of DEGs (1398), far exceeding those in BaP-As(III) vs. BaP (490) and BaP-As(III) vs. CK (89). Notably, the BaP vs. CK group also contained the largest number of unique DEGs (963), which was substantially higher than the unique DEGs in BaP-As(III) vs. BaP (52) and BaP-As(III) vs. CK (17). These results demonstrate that single BaP stress exerted the most profound impact on bacterial gene expression, and the addition of trivalent arsenic did not induce new large-scale differential expression. Meanwhile, 395 DEGs were shared between the BaP-As(III) vs. BaP and BaP vs. CK comparisons, a number significantly higher than that of other pairwise overlaps, further confirming that the introduction of trivalent arsenic primarily modulated the transcription levels of a subset of genes that were already responsive to BaP alone. Furthermore, the BaP-As(III) vs. CK comparison exhibited the lowest number of DEGs, suggesting that under arsenic-BaP combined stress, the bacterial gene expression pattern tended to revert toward that of the control group. This observation implies that trivalent arsenic and BaP may exert more complex interactive effects at the transcriptional regulatory level.

The volcano plots further delineated the direction and statistical significance of differential gene expression across all treatments (Figure 3). Under single BaP stress, 1013 genes were significantly upregulated and 848 were significantly downregulated (Figure 3d), demonstrating that BaP exposure elicits robust bidirectional transcriptional perturbations in the strain. In contrast, the combined arsenic-BaP stress induced only 122 significant DEGs (97 upregulated, 25 downregulated) (Figure 3e), indicating that the addition of As(III) drastically attenuated transcriptional divergence from the control group. When compared with the single BaP treatment, the introduction of trivalent arsenic led to 452 upregulated and 145 downregulated genes (Figure 3f), which further validates that As(III) exerts a measurable regulatory effect on the strain’s BaP degradation capacity.

#### 3.4.2. Functional Enrichment Analysis of Different Treatment Groups

**Figure 4:**
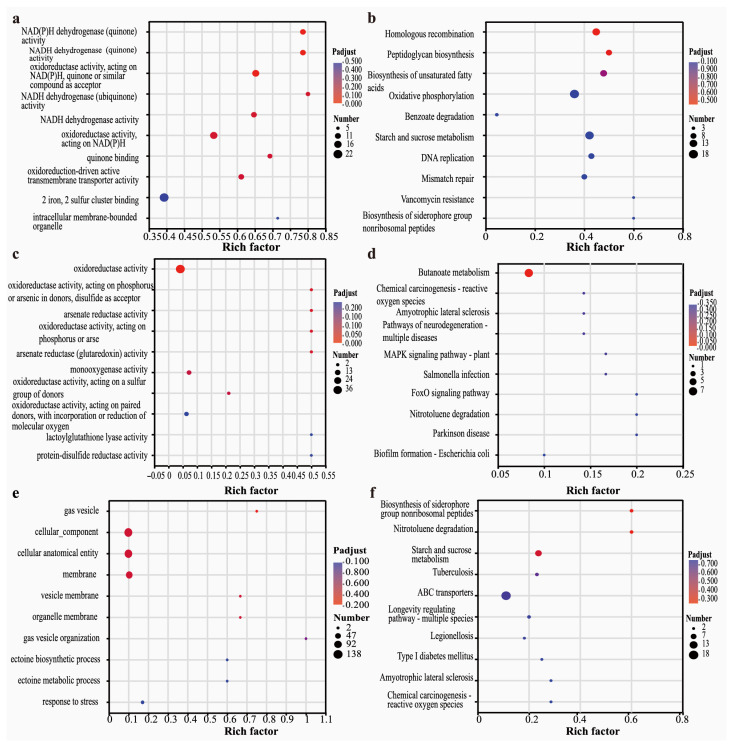
GO and KEGG enrichment analyses of different comparison groups. (**a**) GO enrichment and (**b**) KEGG enrichment between BaP and CK treatments. (**c**) GO enrichment and (**d**) KEGG enrichment between BaP-As(III) and CK treatments. (**e**) GO enrichment and (**f**) KEGG enrichment between BaP-As(III) and BaP treatments. The dot size corresponds to the gene number, and the dot color corresponds to statistical significance. The statistical significance threshold level for all GO and KEGG enrichment analyses was *p* < 0.05 (BH corrected for multiple comparisons).

As shown in Figure 4a, the BaP vs. CK treatment group exhibited significant GO term enrichment in NADPH dehydrogenase (quinone) activity, quinone oxidoreductase activity, iron-sulfur cluster binding, and inner membrane-bound organelles. NADPH-dependent oxidoreductases, as core components of the cytochrome P450 enzyme system, are responsible for the ring opening and hydroxylation of BaP. The enrichment of inner membrane-bound organelles suggests that the degrading enzyme system is predominantly localized on the cell membrane, which mediates transmembrane transport and in situ metabolism of the substrate. For KEGG pathway enrichment (Figure 4b), benzoate degradation, oxidative phosphorylation, homologous recombination, and peptidoglycan biosynthesis were significantly enriched in the BaP vs. CK group. The benzoate degradation pathway serves as a core downstream pathway for PAHs and is directly involved in the stepwise metabolism of PAHs. The oxidative phosphorylation pathway supplies energy for the degradation process, while the enrichment of homologous recombination and peptidoglycan biosynthesis indicates that cells initiate repair mechanisms in response to BaP-induced toxic stress.

In the BaP-As(III) vs. CK treatment group (Figure 4c), GO was significantly enriched in arsenate reductase activity, oxidoreductase activity (acting on phosphorus/arsenic donors), and protein disulfide reductase activity. Protein disulfide reductase is involved in the repair of oxidatively damaged proteins and alleviates oxidative stress under combined stress. Arsenate reductase is the first step in arsenic detoxification by bacteria, reducing As(III) to As(V).

In the BaP-As(III) vs. BaP group, the core GO terms were significantly enriched in gas vesicle formation, membrane component organization, and stress response (Figure 4e). For KEGG pathway analysis (Figure 4f), ABC transporters, siderophore biosynthesis, and nitrotoluene degradation were identified as the significantly enriched pathways. ABC transporters serve as the core carriers for the efflux of trivalent arsenic in bacterial cells and directly mediate arsenic detoxification in bacteria. Notably, the marked enrichment of nitrotoluene degradation may imply a reduced activity of the BaP degradation pathway. Collectively, under single BaP stress, bacteria adopt the activation of BaP metabolic pathways and energy metabolism as their primary response strategies; in contrast, under combined BaP-As(III) stress, the bacterial strain shifts its response toward oxidative stress defense and arsenic detoxification mechanisms due to the toxic effects of arsenic.

#### 3.4.3. Differences in Expression Levels of Core Functional Genes Between Groups

KEGG basic annotation revealed multiple genes involved in BaP degradation (ko00624), including *pcaH/G*, *ndoB*, and genes encoding phthalate 4,5-dioxygenase reductase (gene1006, gene3241). A complete ars operon (*arsR*, *arsD*, *arsA*, *arsB, arsC*) for inorganic arsenic detoxification was also identified. Expression differences were analyzed based on these annotated gene sets.

**Figure 5:**
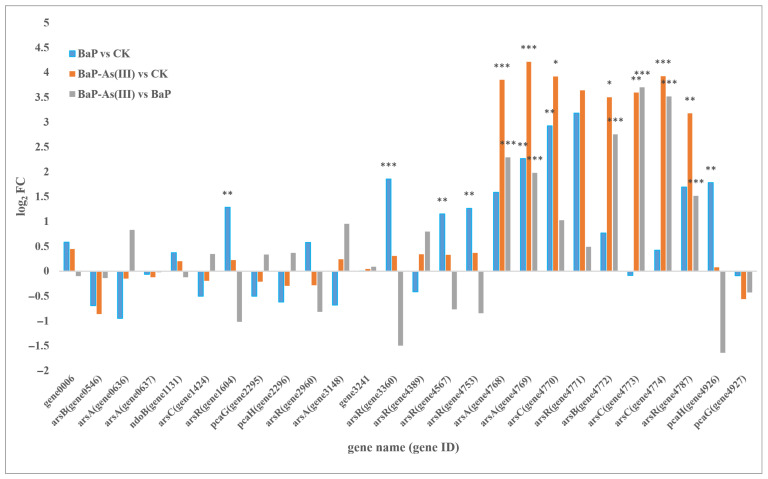
Differences in the expression levels of target genes among different treatment groups. The three treatment groups were compared pairwise, namely BaP vs. CK, BaP-As(III) vs. CK, and BaP-As(III) vs. BaP. The abscissa represents the names of genes related to polycyclic aromatic hydrocarbon metabolism in strain PDS1 annotated by KEGG and genes involved in arsenic detoxification processes in bacteria (if there is no name, the gene ID is used instead). The ordinate is the log_2_FC value. A larger log_2_FC value indicates a greater fold change in the expression of up-regulated genes; a smaller log_2_FC value indicates a greater fold change in the expression of down-regulated genes; a log_2_FC value closer to 0 indicates a smaller fold change in the differential expression of genes. Significance levels: * *p* < 0.05 (significant), ** *p* < 0.01 (highly significant), *** *p* < 0.001 (very highly significant).

Compared with the CK group (Figure 5), the bar chart revealed that, under single BaP stress, genes related to the metal regulatory protein ArsR/SmtB family transcription factors (gene1604, gene3360, gene4567, gene4753), the arsenite efflux transporter metallochaperone ArsD (gene4769), the low-molecular-weight phosphatase family protein ArsC (gene4770), and the β subunit of protocatechuate 3,4-dioxygenase (gene4926) were significantly up-regulated. The gene encoding the α subunit of naphthalene 1,2-dioxygenase (NdoB) and the phthalate 4,5-dioxygenase reductase component gene were non-significantly up-regulated, indicating that the strain initiated. BaP ring-opening degradation. Notably, BaP exposure induced the up-regulation of ArsR/SmtB, implying that PAH toxicity activates the cellular stress response system. The ROS produced under PAH stress can alter cell membrane permeability, damage iron-sulfur cluster–containing enzymes and intracellular metal-binding proteins (e.g., ferritin, zinc finger proteins), and cause metal ion leakage into the cytoplasm [36]. This triggers an intracellular “pseudo-metal overload” state [31], thereby inducing the up-regulated expression of ArsR/SmtB.

As shown in the bar chart of BaP-As(III) vs. CK (Figure 5), genes involved in arsenic detoxification—including the transcriptional regulator (gene4787), arsenical pump-driving ATPase (gene4768), arsenite efflux chaperone ArsD (gene4769), and arsenate reductase-related genes (gene4770, gene4772, gene4773, gene4774)—were significantly upregulated. The gene encoding naphthalene 1,2-dioxygenase α-subunit (gene1131) and phthalate 4,5-dioxygenase reductase genes (gene1006, gene3241) showed non-significant upregulation, whereas protocatechuate 3,4-dioxygenase was non-significantly downregulated. These results indicate that, under dual stress, the strain prioritizes the arsenic response and rapidly activates arsenic detoxification genes, while slightly upregulating BaP upstream degradation genes to alleviate substrate toxicity. This is consistent with the Box–Behnken regression model in Section 3.2.1, which demonstrated that As(III) exerts a stronger effect on BaP removal efficiency than BaP concentration itself.

As shown in the bar chart of BaP-As(III) vs. BaP (Figure 5), arsenic detoxification genes (gene4787, gene4768, gene4769, gene4770, gene4772, gene4773, gene4774) were significantly upregulated under dual stress. The naphthalene 1,2-dioxygenase α-subunit gene (gene1131), phthalate 4,5-dioxygenase reductase gene, and protocatechuate 3,4-dioxygenase genes (gene4926, gene4927) were non-significantly downregulated, whereas gene3241, encoding the 2Fe-2S iron-sulfur cluster–binding protein core to phthalate 4,5-dioxygenase reductase, was up regulated. These results demonstrate that, under combined stress, the strain preferentially responds to the more toxic As(III), resulting in the down regulation of BaP degradation-related genes. These results indicate that, under combined stress, the strain preferentially responds to the more toxic As(III). Nevertheless, the expression of BaP-related genes is not significantly affected, and may even promote the conversion of BaP toward the phthalate pathway.

## 4. Discussion

Polycyclic aromatic hydrocarbons and heavy metals frequently coexist as combined pollutants in the environment, which aggravates ecological risks, endangers human health, and increases challenges for environmental remediation, thus becoming a major public concern. As a green, safe, and efficient strategy, microbial in situ remediation has gradually emerged as a research hotspot. However, according to current reports, only a limited number of strains can simultaneously degrade PAHs and transform heavy metals. In the study by Shi [33], *Rhodococcus* sp. 2021 exhibited high degradation efficiency toward fluorene, a low-molecular-weight PAH, with a degradation rate of 91.18% at a substrate concentration of 200 mg/L, while converting approximately 13% of As(III) to As(V). In the work by Han et al. [37], *Altererythrobacter* sp. H2 was able to reduce Cr(VI) and oxidize As(III), but it mainly degraded fluoranthene and pyrene effectively, with only about 9.5% degradation of 5 mg/L BaP within 7 days. In the study by Peng et al. [38], *Arthrobacter oxydans* DSM 20119 degraded 68.3% of BaP after 7 days of incubation at a substrate concentration of 1 mg/L, and could adsorb Cd(II) but lacked transformation ability. As reported by Kotoky et al. [39], *Bacillus subtilis* SR1 degraded 35% of BaP after 21 days of culture at 2 mM and showed tolerance to Cd(II) but no transformation capacity. *Bacillus megaterium* BB-1 achieved 52.1% degradation of BaP at 10 mg/L after 8 days, and exhibited tolerance to Cu(II) and Cd(II) without transformation function [40]. Cheng Tianzuo constructed a composite microbial consortium using *Klebsiella* sp. CW-D3Ta and *Arthrobacter* sp. SZ-3, which degraded 79% of phenanthrene within 10 days under Cd stress. Most existing studies focus on single substrates, composite microbial communities, or PAH degradation under heavy metal tolerance. In contrast, investigations on bifunctional single strains capable of degrading PAHs—especially high-molecular-weight PAHs—and transforming heavy metals remain scarce. In this study, we found that, under combined BaP-arsenic stress, the *Rhodococcus* sp. PDS1 tolerated 5 mM As(III), and the removal rate of BaP in the liquid reaches 94.12% after 3 weeks of cultivation with a substrate concentration of 10 mg/L. It achieved self-detoxification via arsenic reduction and efflux, and simultaneously converted approximately 11% of extracellular As(III) to As(V), while efficiently removing highly toxic and low bioavailability BaP. These results demonstrate that strain PDS1 has considerable advantages in the remediation of BaP-arsenic combined pollution.

As a toxic signal molecule, As(III) plays a dual dominant role in triggering toxic stress and regulating cellular metabolism. Arsenic detoxification and metabolic pathways in bacteria are mainly classified into four categories [41]: (1) The arsenic resistance (*ars*) system, the most extensively studied arsenic detoxification pathway in bacteria. The *ars* operon confers resistance to inorganic arsenic compounds, with the most common architectures being the three-gene operon *arsRBC* or the five-gene operon *arsRDABC*. (2) Arsenic methylation and organoarsenic detoxification. As(III) is sequentially methylated under the catalysis of ArsM to produce MAs(III), DMAs(III), and even TMAs(III) (gaseous). MAs(III) can be oxidized to MAs(V) by ArsH, reduced back to As(III) by ArsI, or conjugated with 3-phosphoglyceric acid (3PGA) to form 1As3PGA, which is then oxidized to As(V) by ArsJ, thereby reducing toxicity. (3) The arsenite oxidation (*aio/arx*) system. Enzymes such as AioBA/ArxA encoded by operons including *aioAB* and *arxAB* oxidize As(III) to As(V), which is coupled to respiratory energy generation and thus functions as part of energy metabolism while alleviating As(III) toxicity. (4) The arsenate reduction (*arr*) system, which operates mainly in anaerobic respiration. It reduces As(V) to As(III) via enzymes such as ArrAB, using As(V) as the terminal electron acceptor to support energy production. The resulting As(III) can further participate in other metabolic pathways or be extruded from cells.

A complete *arsRDABC* operon was annotated in strain PDS1, which achieves arsenic detoxification through arsenic reduction and efflux. This mechanism is consistent with the arsenic resistance strategy reported in *Rhodococcus* sp. by Kumari [42], Firrincieli [43], and co-workers. Notably, no genes encoding enzymes responsible for intracellular arsenic oxidation were identified in PDS1, yet approximately 11% of As(III) was converted to As(V) in the culture system (Figure 2). The underlying mechanism remains unclear, but several hypotheses can be proposed based on previous studies. (1) Extracellular arsenic oxidation mediated by quinone/flavin electron shuttles. *Shewanella oneidensis* MR-1 secretes riboflavin and flavin mononucleotide (FMN) as electron shuttles to oxidize As(III) to As(V) extracellularly. The cyclic regeneration of flavins enables sustained oxidation, providing a classic model for extracellular electron transfer-driven arsenic oxidation [44]. In the study by Jiang et al. [45], semiquinone radicals generated during microbial or chemical reduction in the humic acid model quinone AQDS (9,10-anthraquinone-2,6-disulfonic acid) act as strong oxidants capable of oxidizing arsenite to arsenate, thereby reducing arsenic toxicity and mobility. (2) Extracellular oxidation mediated by reactive oxygen species (ROS). In the work by Ai et al., algal-bacterial biofilms stimulated by exogenous materials generated substantial extracellular ROS, which directly mediated the oxidation of As(III). (3) Extracellular arsenic oxidation coupled with biofilms and interfacial processes. Fu et al. observed a marked consumption of polysaccharides in bacterial extracellular polymeric substances (EPS) during extracellular oxidation of As(III) to As(V), implying that extracellular polysaccharides may be directly involved in As(III) oxidation [46]. The iron-oxidizing bacterium *Pseudarthrobacter* sp. Fe7 oxidizes As(III) and promotes coprecipitation with biogenic iron minerals [47]. In the thermoacidophilic iron-oxidizing archaeon *Acidianus brierleyi*, Fe(III) produced by Fe(II) oxidation is complexed by EPS, forming a localized highly oxidative microenvironment on the cell surface that efficiently oxidizes As(III) to As(V) and further promotes the formation of iron arsenate precipitates [48]. In addition to the above biological mechanisms, abiotic factors may also contribute to arsenic oxidation. For instance, changes in physicochemical parameters such as pH and dissolved oxygen caused by bacterial growth and metabolism can alter the valence state of arsenic in the system.

The results revealed that, under BaP-only stress, the *pcaH/G* gene and its associated GO term “protocatechuate 3,4-dioxygenase activity” were significantly enriched and upregulated, confirming this enzyme as a core functional component of bacterial PAH degradation. Upon arsenic exposure, the GO term “arsenate reductase activity” linked to *arsC* and other *ars* operon genes was also significantly enriched and upregulated, demonstrating that activation of the complete *ars* operon represents a key mechanism for bacterial arsenic tolerance. Specifically, pentavalent arsenic is reduced to trivalent arsenic by arsenate reductase (ArsC), and As(III) is then extruded from the cell by the arsenite efflux permease (ArsB) encoded by *arsB*. As a membrane transporter, ArsB alone can catalyze As(III) efflux using membrane potential to achieve detoxification, or it can form a stable membrane complex with ArsA—another ars family protein—to enhance As(III) extrusion. Under combined BaP and arsenic stress, both *pcaH/G* and *ars* operon genes were simultaneously upregulated, indicating that bacteria can synergistically activate detoxification and metabolic pathways to enhance their resilience against complex environmental pollution pressures.

**Figure 6:**
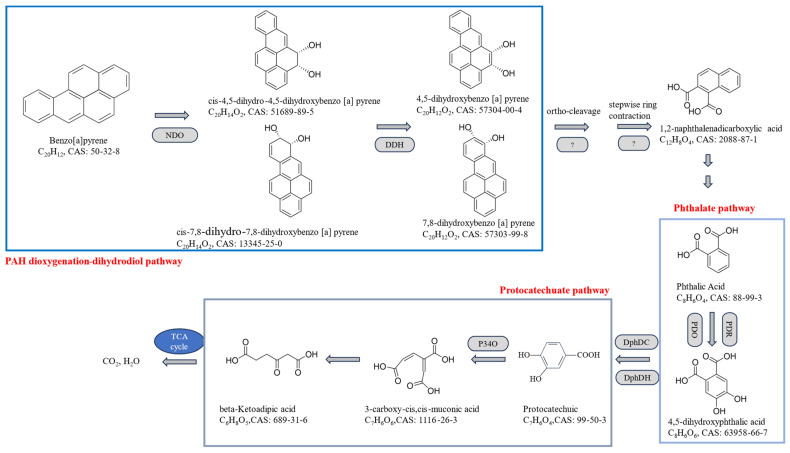
Prediction of BaP degradation pathway based on transcriptome sequencing. NDO: naphthalene 1,2-dioxygenase; DDH: dihydrodiol dehydrogenase; PDO: phthalate 4,5-dioxygenase; PDR: phthalate dioxygenase reductase; DphDC: 4,5-dihydroxyphthalate decarboxylase; DphDH: 4,5-dihydroxyphthalate dehydrogenase; P34O: protocatechuate 3,4-dioxygenase. The question marks in the figure represent that the specific types and quantities of enzymes participating in these pathways have not been fully characterized.

Based on KEGG annotations, the BaP degradation pathway in strain PDS1 may be initiated by initial hydroxylation catalyzed by naphthalene 1,2-dioxygenase. This enzyme introduces two oxygen atoms onto one benzene ring to form cis-dihydrodiol-benzo[a]pyrene, thereby disrupting the chemical stability of BaP and triggering the entire degradation cascade [49] (Figure 6). In *Bacillus* sp. M1, degradation commences with enzymatic attack at the C-9/C-10 or C-7/C-8 positions, yielding cis-4,5-pyrene dihydrodiol [50]. In *Pigmentiphaga kullae* KIT-003, the initial step is mediated by homologous enzymes such as NahAc, leading to the formation of intermediates including 5,6-dihydro-5,6-dihydroxyanthracene-5-carboxylic acid [51]. Following these initial reactions, the pentacyclic structure of BaP is broken down into tri- or bicyclic intermediates for further catabolism, with phthalic acid representing a key branch point. In *Pontibacillus chungwhensis* HN14, BaP is ultimately converted into smaller aromatic compounds such as anthracene, phenanthrene, and naphthalene, which serve as typical precursors entering the phthalic acid pathway [49]. Consistent with findings from Kefir microbial communities, BaP degradation can proceed via both the naphthalene pathway and the phthalic acid pathway [52]. Lower-ring intermediates are further transformed by phthalate dioxygenase into simpler metabolites such as protocatechuic acid. Protocatechuate dioxygenase catalyzes the oxidative ring cleavage of protocatechuic acid, generating products including 2-hydroxy-4-carboxymuconic semialdehyde. This aromatic ring-opening step facilitates entry into the tricarboxylic acid (TCA) cycle, enabling complete mineralization to CO_2_ and H_2_O, while supplying carbon and energy for bacterial growth [53].

In summary, we obtained a dual-functional strain PDS1 capable of degrading benzo(a)pyrene and transforming inorganic arsenic.

## 5. Conclusions

Strain PDS1 is a *Rhodococcus* sp. bacterium isolated from coking plant soil that has been contaminated with polycyclic aromatic hydrocarbons and heavy metals for a long time. This strain not only exhibits a high removal capacity for the HMW-PAH BaP, but also tolerates As(III) at concentrations as high as 5 mM. In this study, RSM was employed to systematically optimize the conditions for BaP degradation by this strain. Meanwhile, transcriptome sequencing was used to comprehensively investigate the mechanisms underlying arsenic tolerance and detoxification, BaP degradation, as well as the synergistic response of strain PDS1 under the combined stress of these two pollutants. Based on transcriptome sequencing analysis combined with physical and chemical property detection, we speculate that strain PDS1 achieves arsenic tolerance and detoxification through intracellular arsenic reduction and efflux, while naphthalene 1,2-dioxygenase and protocatechuate 3,4-dioxygenase mediate the degradation process of benzo(a)pyrene. Furthermore, within the system, a portion of As(III) was transformed into As(V), which is less toxic, more readily mineralized, and has lower bioavailability. These findings provide a theoretical foundation for the practical application of this strain, which shows promising prospects for the in situ bioremediation of soil co-contaminated with polycyclic aromatic hydrocarbons and heavy metals. However, this study still has certain limitations. At present, only transcriptome sequencing analysis has been performed, and the dissection of the degradation mechanism of strain PDS1 and its response mechanism under dual stress remains insufficient. Future research should combine molecular biological approaches to verify the functional genes of the strain and clarify its complete degradation pathway. Meanwhile, the application of this indigenous strain to in situ soil remediation still needs laboratory-scale simulated remediation experiments to explore its feasibility and practical application potential.

## Figures and Tables

**Table 1:** Experimental Factors and Level Coding.

Level	Factor A	Factor B (mg/L)	Factor C (mM)	Factor D (°C)
−1	6	0.5	0	30
0	8	10	2.5	37
1	10	20	5	42

Factor A: pH value; Fator B: BaP concentration (mg/L); Factor C: As(III) concentration (mM); Factor D: temperature (°C).

**Table 2:** Analysis of variance and significance test of Box–Behnken regression model. Results of ANOVA of the response surface experiments.

Source	Sum of Squares	df	F-Value	*p*-Value	Significant
Model	22,898.28	14	45.33	<0.0001	**
A-pH	12.00	1	0.33	0.5733	not significant
B-BaP Con.	335.61	1	9.30	0.0087	**
C-As(III) Con.	13,195.98	1	365.75	<0.0001	**
D-temperature	31.86	1	0.88	0.3633	not significant
AB	797.44	1	22.10	0.0003	**
AC	46.17	1	1.28	0.2770	not significant
AD	789.44	1	21.89	0.0004	**
BC	119.43	1	3.31	0.0903	not significant
BD	203.21	1	5.63	0.0325	*
CD	8.45	1	0.23	0.6360	not significant
A^2^	2340.52	1	64.87	<0.0001	**
B^2^	628.32	1	17.41	0.0009	**
C^2^	5662.30	1	156.94	<0.0001	**
D^2^	305.67	1	8.47	0.0114	*
Residual	505.12	14			
Lack of Fit	457.76	10	3.87	0.1022	not significant
Pure Error	47.35	4			

Significance levels: * *p* < 0.05 (significant), ** *p* < 0.01 (highly significant). R^2^ = 0.9784, adjusted R^2^ (R^2^_adj) = 0.9568, predicted R^2^ (R^2^_pre) = 0.8816, coefficient of variation (C.V.%) = 10.54%. A is pH value, B is BaP concentration, C is As(III) concentration, and D is temperature. AB, AC, AD, BC, BD, and CD represent the interaction terms between pH value and BaP concentration, pH value and As(III) concentration, pH value and temperature, BaP concentration and As(III) concentration, BaP concentration and temperature, and As(III) concentration and temperature, respectively. A^2^, B^2^, C^2^, and D^2^ represent the quadratic terms of pH value, BaP concentration, As(III) concentration, and temperature, respectively.

## Data Availability

The original contributions presented in this study are included in the article. Further inquiries can be directed to the corresponding authors.

## Appendix A

### Appendix A.1. Information of Isolated Strains

**Table A1:** Alignment results of 16S rRNA gene sequences of the strains.

Isolation Number	Description	Per Ident (%)
H1	*Pusillimonas* sp.	98.97
H2	*Alcaligenes* sp.	99.00
H3	*Achromobacterxylosoxidans*	99.65
H4	*Achromobacter xylosoxidans*	99.72
H5	*Achromobacter xylosoxidans*	99.79
H6	*Rhodococcus pyridinivorans*	99.93
H8	*Rhodococcus* sp.	99.72
H9	*Achromobacter ruhlandii*	100
H10	*Achromobacter xylosoxidans*	99.44
H11	*Rhodococcus pyridinivorans*	99.65
H12	*Rhodococcus aetherivorans*	99.60
H13	*Pusillimonas* sp.	100
H15	*Achromobacter denitrificans*	99.58
H16	*Rhodococcus pyridinivorans*	100
H21	*Rhodococcus pyridinivorans*	99.85
H22	*Rhodococcus pyridinivorans*	99.86
H23	*Pseudomonas stutzeri*	100
H25	*Achromobacter denitrificans*	99.5
H26	*Achromobacter denitrificans*	99.51
H27	*Achromobacter denitrificans*	99.57
H28	*Achromobacter denitrificans*	100
H29	*Achromobacter denitrificans*	99.93
H30	*Achromobacter denitrificans*	99.86
H32	*Achromobacter denitrificans*	99.93
H33	*Achromobacter denitrificans*	99.64
H34	*Achromobacter sp.*	99.57
H35	*Achromobacter denitrificans*	99.65
H36	*Micrococcus* sp.	99.78
H37	*Staphylococcus hominis*	99.93
H38	*Bacillus firmus*	100
H39	*Planomicrobium chinense*	100
H42	*Achromobacter denitrificans*	99.71
H44	*Ochrobactrum* sp.	100
H45	*Stenotrophomonas maltophilia*	100
H46	*Pseudomonas aeruginosa*	100
H48	*Stenotrophomonas* sp.	100
H51	*Arthrobacter* sp.	99.93
H52	*Mitsuaria chitosanitabida*	98
H53	*Kocuria Polaris*	99.93
H54	*Rhizobium* sp.	100
H55	*Microbacterium* sp.	99.86
H56	*Brevundimonas* sp.	100
H57	*Gordonia* sp.	100
H58	*Mesorhizobium hankyongi*	100
H59	*Microbacterium oxydans*	99.78
H60	*Microbacterium oxydans*	100
H61	*Methylorubrum aminovorans*	100
H62	*Lysinibacillus fusiformis*	99.72
H63	*Pseudomonas* sp.	99.79
